# Adult medulloblastoma: a case report

**DOI:** 10.1186/s13256-022-03531-3

**Published:** 2022-08-25

**Authors:** Emmanuel Mduma, Adagi Awuor, Emmanuel L. Lugina

**Affiliations:** 1 Department of Clinical Oncology, Muhimbili University of Health and Allied Sciences, Dar es Salaam, Tanzania; 2 Oginga Odinga Teaching and Referral Hospital, Kisumu, Kenya; 3 Ocean Road Cancer Institute, Dar es Salaam, Tanzania; 4 Arusha Lutheran Medical Centre, Arusha, Tanzania

**Keywords:** Case report, Chemotherapy, Craniospinal radiation, Medulloblastoma, Ocean Road Cancer Institute, Posterior cranial fossa

## Abstract

**Background:**

Medulloblastoma is a malignant brain tumor that is common in children but very uncommon in adults, especially those older than 40 years, accounting for less than 1% of all primary brain tumors in adults. Although surgery and radiotherapy play an important role treatment of adult medulloblastoma, the use of chemotherapy is controversial. This is the first instance of adult medulloblastoma at the Ocean Road Cancer Institute in Tanzania.

**Case description:**

We report the case of a 51-year-old female of African ethnicity who was diagnosed with high-risk hemispheric posterior cranial fossa medulloblastoma of classic type with World Health Organization central nervous system grade 4 and Chang stage M0. Immunohistochemistry, reticulin stain, and molecular subtyping could not be done because they were not available. She was treated by subtotal posterior cranial fossa tumor resection followed by adjuvant concurrent chemo-craniospinal radiation and adjuvant chemotherapy.

**Conclusion:**

Even in adults over 50 years old, medulloblastoma should be included in the differential diagnosis of posterior fossa tumor. Adult medulloblastoma is a very rare and very heterogeneous tumor, but it has a good prognosis. Immunohistochemistry and molecular subclustering are difficult to implement in low-income countries such as Tanzania owing to cost. Treatment of adult medulloblastoma is highly heterogeneous among (and even within) facilities. There is no evidence that the extent of resection enhances survival. While craniospinal radiation therapy improves survival, there is controversy about the role of chemotherapy in managing adult MB.

## Introduction

Medulloblastoma (MB) is a highly heterogeneous, aggressive, and invasive malignant embryonic tumor that arises in the cerebellum [1]. It is the most common malignant brain tumor in children, accounting for nearly 20% of all pediatric brain tumors. Conversely, adult MB is extremely rare and accounts for less than 1% of intracranial tumors [2]. Smoll *et al.* showed that MB is 10 times more likely to be diagnosed in children than adults and that male gender is a risk factor for the development of MB in children only but not in adults [3].

The clinical symptoms and signs of MB in adults are associated with the location of the tumor in the posterior fossa, increased intracranial pressure, and/or obstruction of the cerebrospinal fluid pathway leading to headache, dizziness, nausea, ipsilateral cerebellar signs, and ataxia [4].

In adult MB, magnetic resonance imaging (MRI) of the brain shows hypointense T1 and hyper to hypointense T2 lesions. The intensity and extent of contrast improvement on T1 postcontrast imaging vary significantly, hence histopathological examination should be performed to confirm the diagnosis [5].

There are four major histological subtypes of MB with prognostic value: classical (CMB), desmoplastic/nodular (DNMB), large cell anaplasia (LCMB), and extensive nodularity/anaplastic (AMB). The most common histological variant is classic MB in children and adults (70–80%) [5]. Among adults, DNMB has the longest OS and PFS, followed by CMB and AMB with the worst treatment outcome [6].

There are four molecular subgroups of MB with prognostic value: wingless (WNT), sonic hedgehog (SHH), group 3, and group 4 [7]. In a study done by Zhao *et al.*, it was shown that there is a preponderance of SHH-type tumors in adult MB (62%), followed by group 4 tumors (28%) and WNT-activated tumors (10%), with an absence of group 3 cases, suggesting that this subgroup may be restricted to pediatric MB [6]. Among adults, WNT has the longest OS and PFS followed by SHH, while group 4 has the worst prognosis. The age distribution of patients with SHH tumors is concentrated in the range of 20–40 years, whereas group 4 and WNT tumors have a wider distribution of age of onset (18–55 years). DNMBs are mainly found to be of SHH subtype, whereas LCMB/AMBs are only found in the SHH and group 4 subtypes. Classic histology is found in all molecular variants. Male patients predominantly have SHH subtype and group 4 tumors, whereas female patients predominantly have tumors of WNT subtype. SHH tumors tend to have a hemispheric location, and WNT tumors have a lower incidence of fourth ventricular floor involvement. Tumors with classic histology account for the largest proportion of all molecular variants, especially among those of WNT subtype, 95% of which tumors have classic histology. Most DNMB tumors belong to the SHH subtype. Anaplastic MBs and LCMBs are found to be either SHH or group 4 tumors [6]. Whether the histological categorization of MB retains its prognostic value once the molecular subgroup is widely available for all patients has yet to be determined [8].

Chang staging for MB considers tumor size and disease spread to determine low- and high-risk groups. Patients with tumors over 3 cm with unequivocal propagation in the brainstem or beyond the aqueduct of Sylvius and/or foramen magnum noted during surgery or on imaging, or any metastasis outside of the brain parenchyma, are high risk [9]. Adult patients with high-risk MB have lower survival compared with standard-risk patients. However, unlike children, for adults, the prognostic value of the extent of resection is controversial [2]. Metastatic disease at onset is far less common in adults than in children (7% versus 30%) [10]. These differences show that clinical staging of MB may not be as relevant in adults as in children, and the prognosis is likely driven mostly by molecular subgroups and not the extent of the disease [8].

Current management of adult MB is often extrapolated from pediatric protocols and includes maximum safe resections [11], followed by craniospinal radiation therapy (CSRT), with or without concurrent and/or adjuvant chemotherapy depending on clinical risk stratification. Even though the treatment of adult MB is very heterogeneous across (and even within) institutions, a common practice for the last several decades has been to treat patients with complete resection and nonmetastatic dissemination with CSRT alone, whereas patients with incomplete resections and/or metastatic dissemination are treated also with upfront chemotherapy [2]. Over the past two decades, MB survival has been improved by considerable advancements in therapeutic strategies based on clinical risk stratification. However, MB is still associated with a poor outcome overall, and most survivors are left with long-term disabilities secondary to treatment [6].

We report herein the case of a 51-year-old woman with a cerebellum mass who was diagnosed with adult MB. This is the first case to be reported in Tanzania.

## Case presentation

**Fig. 1:**
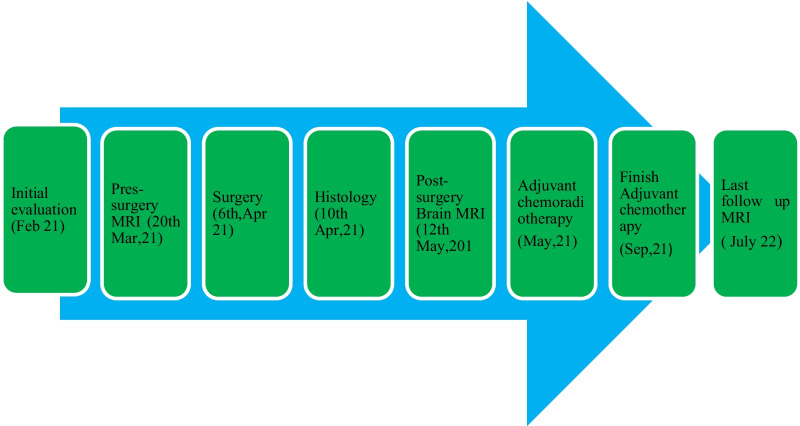
A timeline of important events

**Fig. 2:**
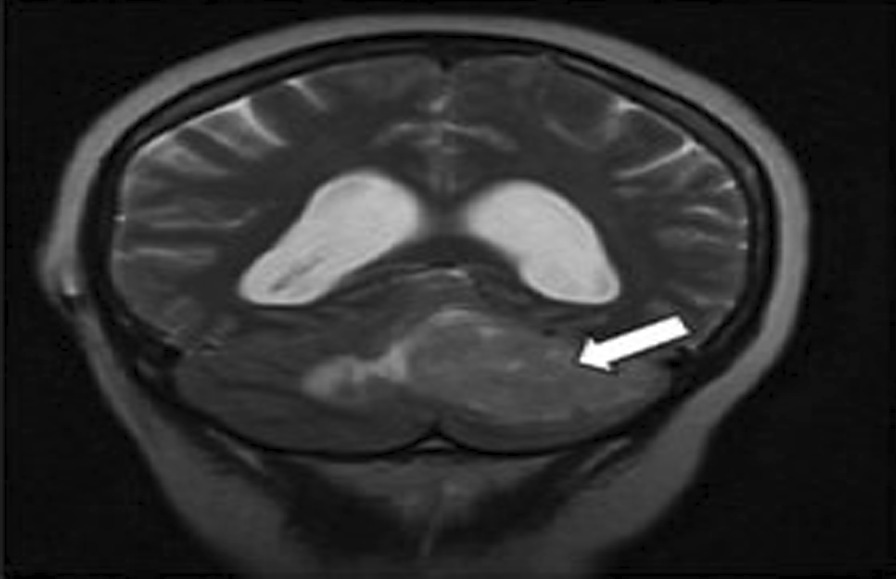
T1W image showing hypointense left lateral posterior cranial fossa lesion as indicated by the arrow

A 51-year-old female of African ethnicity was referred to our oncology center for adjuvant therapy. She had a chief complaint of occipital headache which was associated with dizziness and projectile vomiting for over a year. She had no history of chronic illness. She had three children, and all childbirths were done by cesarean section. She had no previous history of blood transfusion. She had no history of malignancy or cancer within her family. She worked as a nurse. She was married. She denied a history of use of tobacco, alcohol, or other drugs. All vitals were within normal limits. Physical examination was essentially normal. Neurological findings showed that all cranial nerves were within normal limits, and there was no motor or sensory deficit, but she had poor coordination of movement. Basic laboratory investigations were done and included: complete blood count (WBC-4.92 × 10^3^/uL, Hb-12.3 g/dl and platelet-165.0 × 10^3^/uL), urea (5.5 mmol/L), serum creatinine (85.19 micromol/L), alanine aminotransferase (23.1 IU/L), Aspartate aminotransferase (30.1 IU/L), total bilirubin (0.19 mg/dl), and albumin (38.45 g/L) essentially normal with negative human immunodeficiency virus (HIV) test. Brain MRI was conducted (Fig. 1) and showed a left posterior fossa mass, which had an iso-hypointense appearance on T1-weighted images, an inhomogeneously enhanced appearance on T1-weighted contrast images and iso-hyperintensity on T2-weighted images (Fig. 2).

A ventriculoperitoneal shunt procedure was conducted before surgery to overcome hydrocephalus. Craniotomy for the posterior fossa tumor was done (Fig. 1). The patient was placed in prone position with her head supported on the Mayfield head holder. A posterior midline suboccipital approach was used. Through the displaced left cerebellar, a grayish solid mass, with an undefined border, was identified and dissected carefully with cauterization of the feeder vessels. Subtotal tumor resection (STR) was done, and hemostasis was achieved. Dura closure was achieved with a dura patch, and the bone flap was returned. Wound closure was done in layers. A histopathology sample from the mass was obtained. The postsurgery phase was uneventful.

**Fig. 3:**
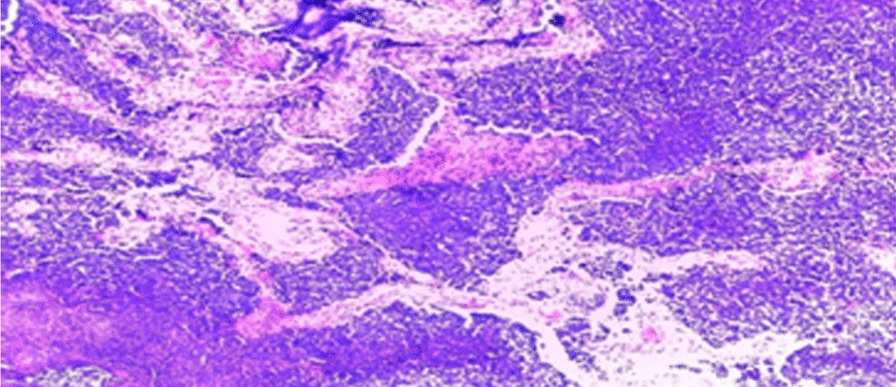
Diffuse and solid sheets with focal areas of nodularity, but desmoplasia was not evident

**Fig. 4:**
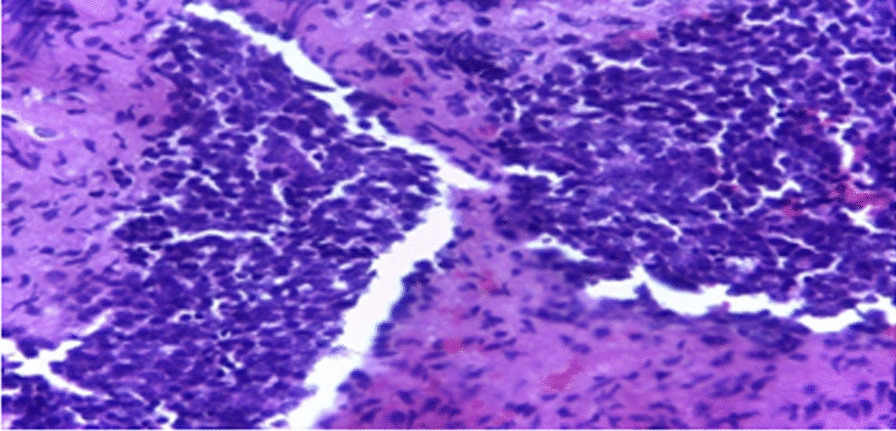
Small round cells with dense nuclear chromatin, scant and indistinct cytoplasm

Histopathology revealed cells with minimal cytoplasm and dense basophilic nuclei present in diffuse sheets in keeping with MB of classic type (CMB) with WHO CNS grade 4 (Figs. 3 and 4). Reticulin staining was not done because it was not available. Immunohistochemical analysis and molecular subgroup were also not performed because they were not available.

We did not carry out CSF analysis to investigate the leptomeningeal spread of the tumor due to concerns about transtentorial herniation. We conducted MRI of the brain and spine at 1 month after surgery (Fig. 1), which revealed a residual posterior fossa tumor without leptomeningeal spread. We stratified the tumor as high risk, and the staging was modified to Chang stage M0. We treated the patient with adjuvant concurrent chemoradiotherapy and adjuvant chemotherapy.

**Fig. 5:**
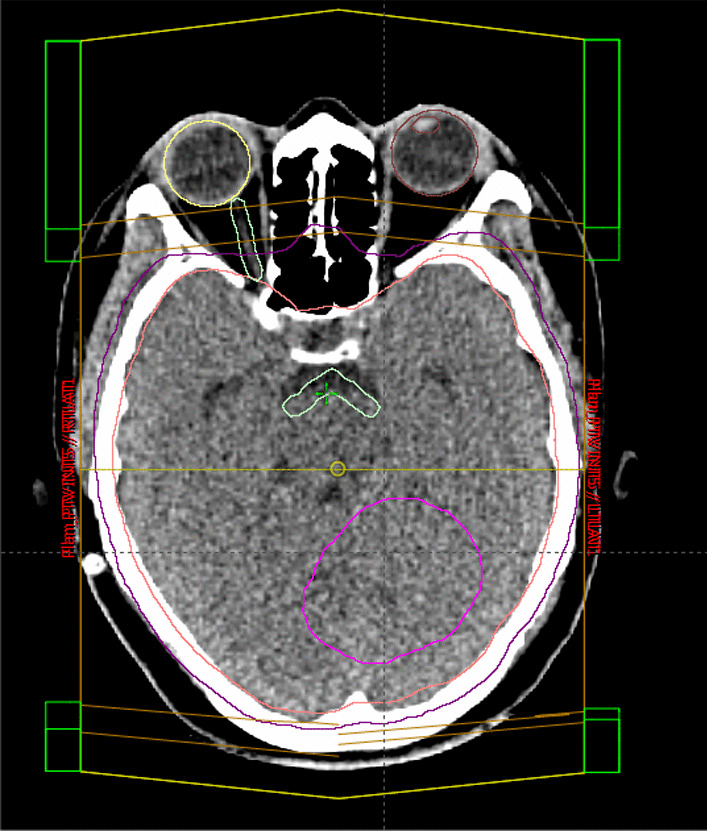
Whole-brain lateral fields

**Fig. 6:**
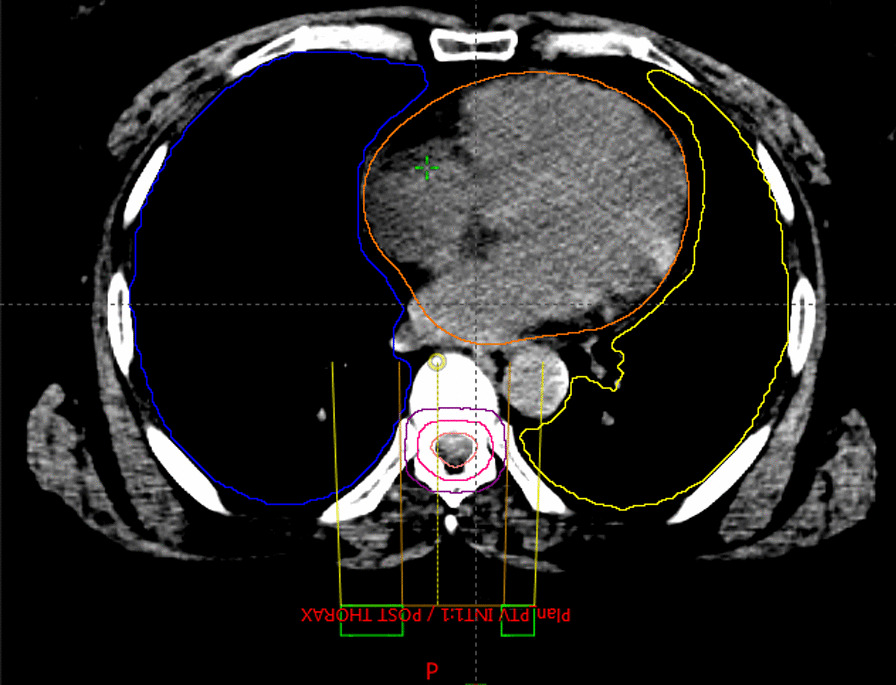
Posterior radiotherapy field to the spinal cord

She was simulated by using CT–Simulator in supine position. The headrest and a thermoplastic head and neck mask were used to restrain the head. Contouring was done for the target and the organs at risk. Laterally parallel opposed fields with photon energy of 6 MV were used to treat the brain (Fig. 5), and two direct posterior adjacent fields were used to treat the spinal cord (Fig. 6). Collimator rotation was used to match the cranial and spinal fields, and a feathering maneuver was used in the two adjusted spine fields to avoid hotspots. The radiotherapy technique was 3D conformal radiotherapy (3DCRT) using a linear accelerator (LINAC). The patient underwent two phases of treatment. The first phase was craniospinal radiation therapy (CSRT) whereby 36 Gy in 20 fractions was delivered, and the second phase was the posterior cranial fossa boost, whereby a boost dose of 18 Gy in 10 fractions was delivered. A weekly dose of 2 mg vincristine was administered intravenously with radiation therapy. She tolerated the treatment very well apart from fatigue, nausea, and dizziness.

Upon completion of radiotherapy, she was put on a monthly course of adjuvant intravenous chemotherapy (etoposide 50 mg/m^2^ D1–D4 and cisplatin 20 mg/m^2^ D1–D4) for a total of six cycles from June to September 2021.

**Fig. 7:**
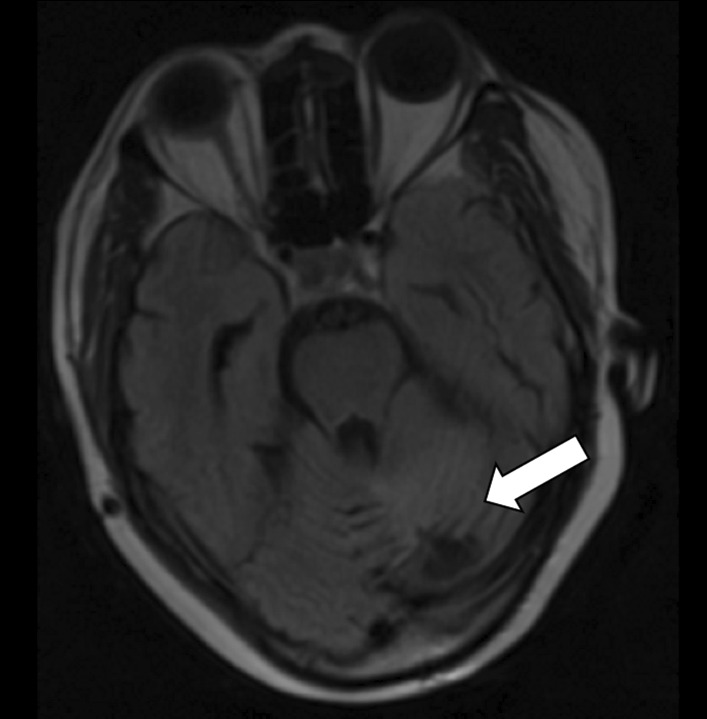
T1W brain MRI 9 months after finishing treatment showing a significant reduction in the posterior cranial fossa lesion as indicated by the arrow

Post-treatment brain MRI showed a significant decrease in the posterior fossa tumor (Fig. 7).

She is currently doing well, apart from mild dizziness.

## Discussion

We report the case of a 51-year-old woman with a high-risk hemispheric posterior cranial fossa MB of classic type (CMB) with WHO CNS grade 4 and Chang stage M0. MB is extremely rare in adults, accounting for less than 1% of intracranial tumors [2]. This is the first documented case of adult MB to be reported in Sub-Saharan Africa.

The tumor in this case report was hemispheric and located in the left side posterior cranial fossa. In adults, MB most frequently involves the cerebellar hemisphere; thus, it is usually more lateral than the midline vermis, which commonly occurs in the pediatric population [12]. Poorer PFS and OS have been observed among adults with tumors located in the midline than in those with tumors located in the hemispheres [6].

The patient had a classical variant of MB, but immunohistochemical analysis and molecular subgroup were not performed because they were not available. Immunohistochemistry and molecular subgroups are costly and difficult to perform in low-income countries such as Tanzania [13]. Moreover, reticulin staining could also not be carried out because it was not available. Reticulin staining could help to rule out the DNMB subtype, which is the most common histologic associated with hemispheric adult MB. The age, hemispheric location, and good prognosis in this case suggest a SHH subtype.

The patient was treated by subtotal resection (STR), and a ventriculoperitoneal (VP) shunt was inserted. According to Majd *et al.*, about 20% of adult MB patients required VP shunts to relieve hydrocephalus [8]. Maximum safe resection has also been the goal of surgical resection in adults [11]. Nayak *et al.* evaluated the survival benefit of extent of resection (gross tumor resection or GTR versus STR) in pediatric MB. Interestingly, no significant survival benefit was found with greater extent of resection for patients with WNT, SHH, or group 3 tumors, questioning the benefit of “second-look” surgeries to remove small residual portions of tumor given the potential for neurological deficits associated with posterior fossa tumor resection [13].

The patient was treated with CSRT concurrently with chemotherapy and adjuvant chemotherapy, as reported previously [14]. CSRT is a favorable prognostic factor, according to a large meta-analysis of literature in adult MB patients [15]. MB is a chemotherapy-sensitive tumor, and chemotherapy plays an established role in treating pediatric MB patients, especially those younger than 3 years of age, to delay radiotherapy [16]. Exact cytotoxic agents and timing of upfront chemotherapy with CSRT (before and after RT) have been controversial in both the pediatric and adult populations, although at present, adjuvant chemotherapy is favored in standard practice [17].

It remains unknown whether chemotherapy plays a role in adult MB. In 2012, Frederick *et al.* published a prospective study in which they followed 70 adult patients with MB treated with postoperative CSRT that was followed (in 49 of these patients) by chemotherapy according to a pediatric protocol. They did not find any prognostic difference between the groups treated with or without chemotherapy [18]. In another retrospective analysis of the national database, it was shown that concurrent postoperative chemotherapy and CSRT are associated with superior survival for patients with adult MB compared with radiotherapy alone [19].

This patient has a good prognosis despite STR. The Surveillance, Epidemiology and End Results (SEER) database with 454 patients with adult MB showed relative survival rates at 2, 5, and 10 years of 79.9%, 64.9%, and 52.1%, respectively [20].

## Conclusion

Even in adults over 50 years old, MB should be included in the differential diagnosis of posterior fossa tumor. Adult MB is a very rare tumor, but with a relatively good prognosis. Immunohistochemistry and molecular subgroup analyses are challenging to implement in low-income countries such as Tanzania because of the cost. Treatment for adult MB is highly heterogeneous between institutions. There is no evidence that the extent of resection enhances survival. While CSRT improves survival, there is controversy about the role of chemotherapy in the management of MB.

## Data Availability

Not applicable.

## Abbreviations

ECOG: Eastern Cooperative Oncology Group
CNS: Central nervous system
CSRT: Craniospinal radiation therapy
GCS: Glasgow Coma Scale
LINAC: Linear accelerator
MB: Medulloblastoma
ORCI: Ocean Road Cancer Institute
OS: Overall survival
RT: Radiotherapy
VP: Ventriculoperitoneal
PFS: Progression-free survival
CSF: Cerebrospinal fluid
GTR: Gross tumor resection
STR: Subtotal tumor resection
3DCT: 3D conformal radiotherapy
